# Dissonance in the discourse of the duration of diabetes: A mixed methods study of patient perceptions and clinical practice

**DOI:** 10.1111/hex.13245

**Published:** 2021-05-05

**Authors:** Christy J. W. Ledford, Stephanie T. Fulleborn, Jeremy T. Jackson, Tyler Rogers, Haroon Samar

**Affiliations:** ^1^ Department of Family Medicine Uniformed Services University of the Health Sciences Bethesda MD USA; ^2^ Eglin Family Medicine Residency Eglin Air Force Base Eglin FL USA; ^3^ Military Primary Care Research Network Department of Family Medicine Henry M. Jackson Foundation Uniformed Services University of the Health Sciences Bethesda MD USA; ^4^ Department of Family Medicine Madigan Army Medical Center Tacoma WA USA

**Keywords:** diabetes remission, mixed methods, prediabetes, type 2 diabetes mellitus

## Abstract

**Background:**

Remission of diabetes can be rewarding for patients and physicians, but there is limited study of how patients perceive the timeline of a disease along the continuum of glycaemic control.

**Objective:**

To explore how patients perceive the timeline of diabetes along the continuum of glycaemic control and their goals of care and to identify whether family physicians communicate the principles of regression and remission of diabetes.

**Design:**

Mixed methods approach of qualitative semi‐structured interviews with purposive sampling followed by cross‐sectional survey of physicians.

**Participants:**

Thirty‐three patients living with prediabetes (preDM) or type 2 diabetes mellitus (T2DM) at medical centres in Georgia and Nevada; and 387 family physicians providing primary care within the same health system.

**Results:**

Patients described two timelines of diabetes: as a *lifelong condition* or as a condition that can be *cured*. Patients who perceived a lifelong condition described five treatment goals: *reducing glucose‐related laboratory values, losing weight, reducing medication, preventing treatment intensification and avoiding complications*. For patients who perceived diabetes as a disease with an end, the goal of care was to *achieve normoglycaemia*. In response to patient vignettes that described potential cases of remission and regression, 38.2% of physician respondents would still communicate that a patient has preDM and 94.6% would tell the patient that he still had diabetes.

**Conclusions:**

Most physicians here exhibited reluctance to communicate remission or regression in patient care. Yet, patients describe two different potential timelines, including a subset who expect their diabetes can be ‘cured’. Physicians should incorporate shared decision making to create a shared mental model of diabetes and its potential outcomes with patients.

**Patient or Public Contribution:**

In this mixed methods study, as patients participated in the qualitative phase of this study, we asked patients to tell us what additional questions we should ask in subsequent interviews. Data from this qualitative phase informed the design and interpretation of the quantitative phase with physician participants.

## 1 INTRODUCTION

In the past decade, research has demonstrated the potential of the remission of type 2 diabetes mellitus (T2DM).^1^, ^2^, ^3^, ^4^, ^5^, ^6^, ^7^ In the 2009 consensus statement, the American Diabetes Association (ADA) defined remission as serum glucose values below the diabetic range without aid of continuing pharmacologic or surgical treatment.^8^ Blood glucose concentrations exist across a continuum of glucose tolerance from normoglycaemia to T2DM (hyperglycaemia), with prediabetes (preDM) labelling a liminal state of hyperglycaemia just below the diabetes threshold.^9^, ^10^ ADA cautions that preDM should not be viewed as a clinical entity but as a risk factor for diabetes and cardiovascular disease.^11^ Studies demonstrate that this ‘continuum of risk is curvilinear; as A1C rises, the diabetes risk rises disproportionately’.^12^

Recent evidence reveals that preDM can be reversed to normoglycaemia through significant weight loss.^13^, ^14^, ^15^, ^16^ In the Look AHEAD (Action for Health for Diabetes) trial, intensive lifestyle intervention showed significantly greater remission of T2DM in comparison with the diabetes support and education group.^7^ Moreover, patients with T2DM who completed and maintained extensive weight loss of at least 15 kg have experienced prolonged remission of diabetes to either preDM or normoglycaemia.^3^, ^17^, ^18^, ^19^, ^20^, ^21^, ^22^ Evidence for remission of T2DM is best documented in metabolic surgery patients. One meta‐analysis showed that 2 years after metabolic surgery, remission was seen in 63% of patients.^23^

Since publication of the Diabetes Prevention Program (DPP), physicians have been encouraged to counsel patients with preDM regarding effective strategies to decrease the risk of cardiovascular disease and progression to T2DM.^24^ With these goals in mind, it is important to understand the role of goal setting when physicians discuss the diagnosis of preDM or T2DM and the continuum of glucose tolerance. From the time of diagnosis, physicians need to aim to achieve a shared meaningful diagnosis, in which the patient, the physician and the healthcare team have a shared mental model of the diagnosis and resulting goals of care.^25^ Goal‐setting, the sharing of realistic health and well‐being goals by physicians and patients, is core to the theory and effective practice of personalized care planning and seen as particularly important for patients with multiple chronic and long‐term health conditions.^26^ Additionally, shared decision making with goal setting and motivational interviewing results in improvements in patients’ knowledge regarding their diagnosis, the perception of risk and more confident decisions in their care.^27^ Specifically along the continuum of glucose tolerance, literature suggests that informing patients of their diagnosis of preDM represents an opportunity to set goals and motivate patients early in the continuum of glucose tolerance.^5^, ^7^ This is important because the literature also suggests that improved clinician communication with patients can result in return to normal glucose regulation in patients with preDM.^28^ Goals, such as remission of T2DM and regression of preDM, can be empowering for patients and can serve as a tool for family physicians to use to inspire patients.^29^, ^30^ When patients achieve remission of T2DM or regression of preDM, not only does the patient experience positive health outcomes, it can result in decreased costs to healthcare systems.^31^, ^32^, ^33^ Research regarding the ‘cure’ or ‘reversal’ of T2DM was the number one priority of patients in a national survey.^34^

However, it is unclear how physicians recognize remission of T2DM and regression of preDM—acknowledging that a trial of lifestyle modification has resulted in reduced laboratory values, signifying the absence of disease. T2DM, specifically, is widely regarded among physicians as a lifelong disease. Peer‐reviewed literature has framed patient beliefs that T2DM can be cured as evidence that patients have unrealistic expectations of treatment^35^ and that ‘providers should educate patients on the natural history of diabetes’.^36^

The present study first explored how patients perceive the timeline of a disease along the continuum of glycaemic control (to include both preDM and T2DM) and how they describe the goals of their care. Building on the qualitative findings from patients, we then investigated clinical practice to identify if family physicians communicate the principles of regression and remission of preDM and T2DM along the continuum of glucose tolerance.

## 2 METHODS

### 2.1 Phase 1: Patient perceptions through a qualitative approach

Upon Institutional Review Board approval, participants were recruited from a federally funded mixed method study aimed at developing physician training on diabetes care. Using a respondent pool from patient survey data (N = 1025), research coordinators mailed patients information letters to inform them of the interview study and to instruct them how to volunteer to participate. From September 2016 to March 2017, patients volunteered either via telephone or in person to research coordinators embedded in the clinics. A total of 33 interview participants volunteered before we stopped accepting volunteers. We used stratified purposeful sampling,^37^ aiming to illustrate potential differences between patients living with preDM and T2DM. Inclusion criteria included age (25‐65 years old), diagnosis (T2DM or preDM, as notated with an International Classification of Diseases code in their electronic medical record) and enrolment at one of two medical centres in distinct regions of the United States. One medical centre was in Augusta, Georgia, within a geographic region with a high prevalence of diabetes.^38^ The second medical centre was in Las Vegas, Nevada.

Table 1 provides information about individual characteristics of the interview sample. Three variables were collected from the medical record: age, sex and diagnosis. To collect information about race and ethnicity before audio recording began, interviewers asked patients an open‐ended question: How do you describe your race and ethnicity? Treatment modality was also collected during the interview.

**TABLE 1:** Interview sample (patient) characteristics

Mean age (n = 33)		55.64
Race/ethnicity^a^ (n = 33)		
White/Caucasian	14	42.4%
Black/African American	10	30.3%
Asian American (including Thai and Filipino)	4	12.1%
Hispanic	2	6.1%
Mixed race	3	9.1%
Sex (n = 33)		
Male	17	51.5%
Female	16	48.5%
Diagnosis (n = 33)		
Prediabetes	11	33.3%
Type 2 diabetes mellitus	22	66.7%
Treatment modality^a^ (n = 33)		
Oral medication	16	48.5%
Oral and insulin	5	15.2%
None	12	36.4%

^a^ Race/ethnicity and treatment modality were self‐reported at the time of interview.

The principal investigator (CJWL), an expert trained in qualitative and mixed methods, wrote the interview guide in collaboration with an external qualitative methods expert (see Box 1 for the interview guide). The interview guide was designed to elicit patient experiences of their diagnosis conversations with providers and the resulting behaviour changes they made after a diagnosis. The guide was informed by the survey study we previously conducted with this population,^39^, ^40^ which included the Diabetes Illness Representation Questionnaire and its five dimensions: impact, illness coherence, timeline (chronicity), severity and personal relevance.^41^ The final question in the interview guide asked patients to suggest additional questions for subsequent interviews: ‘What else should I have asked about your experience?’ After the first two sets of three interviews, the team discussed the interview guide and changes recommended by patients. Rather than additional questions, patient recommendations led to wording changes in the guide and alterations to the order of questions.

BOX 1 **Interview guide**

Tell me about the first time your provider talked to you about diabetes (or prediabetes).
What was your first *impression* of diabetes (or prediabetes)?
  - What was your first thought at the moment of diagnosis? Why did you think this?
What do you think *cause*d your diabetes?
  Why do you think it started when it did?
How *severe* is your diabetes (or prediabetes)?
  (Probe: Has a provider ever described how severe your disease is?)
How does diabetes *impact* your life?
  (Probes: physical, economic, relational, mental (stress) impacts)
What are your *goals* for treating the disease?
  Tell me about how you’ve treated your diabetes (or prediabetes) and how it’s changed through the years.
  What are the most important results you hope to receive from treatment?
  Did you start with changing diet and exercise? Why did this or why did this not work?
  (If on medication or insulin) When did a physician first mention treating your diabetes (or prediabetes) with medication? Did this prompt you to consider changing your diet and exercise instead?
Do you expect your diabetes (or prediabetes) to last a long *time*?
  Do you expect the diagnosis to change?
  (Probe: source of this information)
What do you *fear* most about diabetes (or prediabetes)?
  (Probe: short term versus long term fears/effects)

CJWL trained one clinically embedded research assistant at each clinical location on the interview guide. Research assistants (RA) observed CJWL conduct the first three interviews at each site. CJWL then observed the RAs conduct two interviews at each site. Throughout this process, verification strategies were used across the design to promote rigour, including memo‐keeping and maintaining methodological coherence/congruence during the process.^42^

To decrease participant burden and maintain privacy, interviews were conducted at patients’ primary care centres. Before each interview, the interviewer completed the informed consent process with the patient. Each interview was audio‐recorded and transcribed verbatim. CJWL and two RAs collected data from October 2016 to April 2017. Interviews lasted about 1 hour and resulted in more than 1732 pages of data.

As this was a secondary analysis, the analytical process was not done concurrently with data collection and instead completed once all data had been collected.^43^, ^44^ In phase one of the analysis, CJWL and RAs along with the clinical research coordinator met multiple times to become immersed in the data and discuss patient perceptions and goals for diabetes care. The four researchers then identified and segmented data that reflected these constructs by analysing half of the interview transcripts to conduct a preliminary thematic analysis and identify potential themes for each research inquiry. In the second phase, CJWL sought to validate these themes by reviewing the analysis conducted on the segmented text while also conducting axial coding to define each theme's characteristics. In the third phase, one RA then used the finalized codebook to analyse the second half of the interviews. This provided an additional opportunity to ensure rigour by further validating the themes, identify those most saturated for final presentation and confirm the thematic characteristics.

### 2.2 Phase 2: Physician practice through a quantitative approach

Following the analysis of the qualitative data, part of the research team (three family physicians and one communication scientist) wrote case scenarios followed by multiple‐choice questions for participants to choose the single best answer. Using ADA guidelines^45^ as a framework, the case questions were designed to assess how physicians would communicate potential regression or remission to a patient. The survey questions were part of a larger cross‐sectional omnibus survey conducted by the Clinical Investigations Committee of the Uniformed Services Academy of Family Physicians (USAFP). Patients from the first phase of this study receive care from family physicians who are members of this organization. After initial survey question development, the USAFP Clinical Investigations Committee evaluated questions for consistency with the overall subproject aim, readability and existing evidence of reliability and validity. Pretesting, conducted with family physicians who were not included in the sampling frame, evaluated questions for flow, timing and readability. Box 2 presents the case questions.

BOX 2 **Cases and case questions presented in survey**

**Table:** 

Case 1: prediabetes	George Curry, a 51 yo male, presents to clinic for follow‐up laboratory results. His current vital signs are BP: 127/ 78 and BMI: 26. He has a history of hypertension. Haemoglobin A1c: 5.8; fasting glucose: 115 Lipid panel: Total Chol: 198; HDL: 48; Triglycerides: 115
	What best summarizes how you would explain the lab results to Curry? “Your lab results indicate… A. … the *risk factor* of prediabetes” B. … you have prediabetes.” C. … you *are at risk* for diabetes.” D. I would not use the words prediabetes or diabetes to explain the results.
	When Curry returns to the clinic in 6 months for a follow‐up appointment with you, he reports that he has successfully changed his diet and increased his physical activity. His only active prescription is Lisinopril for his hypertension. His current vital signs are BP: 124/ 79 and BMI: 24.5. Haemoglobin A1c: 5.2; fasting glucose: 99 Total Chol: 178; HDL: 51; triglycerides: 102
	What best summarizes how you would explain the new lab results to Curry? “Your lab results indicate… A. … the *risk factor* of prediabetes” B. … you have prediabetes.” C. … you *are at risk* for diabetes.” D. I would not use the words prediabetes or diabetes to explain the results.
Case 2: Type 2 diabetes	Kevin Williams, a 54 yo male, presents to clinic for follow‐up laboratory results. His current vital signs are BP: 131/ 88 and BMI: 29. He has a history of hypertension. Haemoglobin A1c: 7.1; fasting glucose: 155 Lipid panel TC: 231; HDL: 35; triglycerides: 174
	What best summarizes how you would explain the lab results to Williams? “Your lab results indicate… A. … the *risk factor* of prediabetes” B. … you *are at risk* for diabetes.” C. … you have diabetes.” D. I would not use the words prediabetes or diabetes to explain the results.
	When Williams returns to the clinic in 6 months for his follow‐up appointment with you, he reports that he has changed his diet and increased his physical activity. His only active prescription is Lisinopril for his hypertension. His current vital signs are BP: 130/82 and BMI: 28.5. Haemoglobin A1c: 6.2; fasting glucose: 124 Lipid panel TC: 195; HDL: 43; triglycerides: 145
	What best summarizes how you would explain the new lab results to Williams? “Your lab results indicate… A. … the *risk factor* of prediabetes” B. … you *are at risk* for diabetes.” C. … you have diabetes.” D. I would not use the words prediabetes or diabetes to explain the results.

The sampling frame included all registered attendees of the annual USAFP scientific assembly. Data were collected anonymously in March 2019 from the start date of the USAFP Annual Meeting through 14 days after the end of the conference. Data were anonymously collected online from participants at the meeting via a link within the USAFP conference mobile application. There was one live session presentation of Omnibus Survey questions and two subsequent conference announcements within the mobile application encouraging survey participation. Three post‐conference email survey invitations were sent to registered conference attendees via their listed registration email addresses.

Of 532 registered conference attendees, 387 attendees (72.7% response rate) responded to the survey. We excluded 65 responses that did not answer the questions from this section of the omnibus. Since this is a study of clinical practice, we also excluded an additional 38 responses that were current medical students or were missing data for year of medical school graduation and year of residency graduation. Therefore, 284 responses are included in analysis. See Table 2 for respondent demographics.

**TABLE 2:** Survey participant (physician) characteristics

Gender (n = 284)	
Male	179 (63%)
Female	105 (37%)
Practice setting (n = 282)	
Academic	133 (46.8%)
Non‐academic^a^	149 (52.5%)
Percentage of time spent in clinical care	Mean 54.68 (SD 31.99)
Number of year of practice	Mean 10.89 (SD 8.24)

^a^ Includes the following practice settings: outpatient family health clinic, family health clinic with inpatient duties or obstetric duties, urgent or acute care clinic, inpatient only and “other”

## 3 RESULTS

### 3.1 Phase 1: Patient perceptions through a qualitative approach

The first inquiry identified the patient perceptions of the duration of the disease. Patients described the two different timelines of diabetes: for some patients, diabetes is a chronic, *lifelong condition*, whereas for a second set of patients, diabetes is a condition that can be *cured* or reversed. The second inquiry sought to identify patient perceptions of the goals of diabetes care. Patients who perceived diabetes as a lifelong condition described five treatment goals: *reducing glucose‐related laboratory values, losing weight, reducing medication, preventing treatment intensification and avoiding complications*. For patients who perceived diabetes as a disease with an end, the goal of care was to *achieve normoglycaemia*. Themes are illustrated using patients’ narratives with thematic characteristics italicized.

#### 3.1.1 TIMELINE 1: ‘We'll have to live with it for the rest of our lives’

Some patients recognized the permanence of the condition at the point of diagnosis. One patient reported, ‘something like [diabetes], that's a life‐changing condition… I know it's not cancer or anything, but this is something we'll have to live with for the rest of our lives’. (Patient 2, T2DM) Yet, other patients described a process of acceptance: they did not accept its permanence until after failed attempts at treatment or lifestyle change.

It’s been eleven years…Early on I thought, okay, we can get over this, and you can get better. And then you’d struggle and fail, and it just seemed like… you know, ‘cause…I guess this is a way to put it. When you have a cold, you take some medicine. You take your antibiotics and then you’re fine. Like and you stop taking the medicine and you’re fine. That’s not diabetes… it’s not something that you just get better from. (Patient 32, T2DM)

Another patient connected this acceptance process to a point in the disease process. ‘It's still early in the disease, if you diet and exercise, you can reverse it. And then I know I’ve said to people, well, you know, I’ve gotten to the point where it's irreversible, and some people have said, oh, no, no, no. You know, if you diet and exercise, you can reverse. But there's certain things… certain consequences that just are irreversible’. (Patient 17, T2DM)

For these patients, a lifelong condition required lifelong attention. One patient described, ‘It's an ongoing thing. You gotta continue working on counting your calories, counting your fat grams, getting your steps in because it doesn't end here. It's about your lifestyle change. And that's what it's all about, so it never stops’. (Patient 28, T2DM)

Patients specified five *treatment goals* that were medically related: *reducing glucose‐related laboratory values, losing weight, reducing medication, preventing treatment intensification and avoiding complications*. Patients repeatedly described the numerical targets of diabetes. One patient wanted to ‘just get on track and get these numbers where they need to be’. (Patient 32, T2DM) Patients described weights, laboratory values and calorie and carb counts all in numerical terms. Patients had specific knowledge and targets about the A1C laboratory value. This patient was encouraged by his progress: ‘I’m on the plan, and my numbers dropped from 10.6 [date] to present, 7.5. So we're not there yet, but we're making strides’. (Patient 28, T2DM) Another patient summarized her goals: ‘I’d love to stick with a reasonable diet as far as my carbs and my calories and my fat grams and all that. I would love to exercise thirty minutes a day for three days a week, as a minimum. Those are my two goals right now. If I could do that, I know I’d be in pretty good shape’. (Patient 17, T2DM)

Patients aimed to *avoid treatment intensification*. For patients diagnosed with T2DM, patients repeatedly talked about the treatment intensification of insulin. One patient admitted, ‘my real fear of diabetes is if I get so bad that I have to start taking the insulin’. (Patient 10, T2DM). For patients living with preDM, patients described avoiding onset of T2DM within the context of avoiding the need for medication at all. This patient connected her goal to delay onset of T2DM to avoiding insulin treatment: ‘I have a fear of needles… I need to get out there and work out because I don't want to be diabetic. So if I’m in a pre‐stage, let me stay there. Who wants to poke themselves? If you know somebody, let me see. I do not want to’. (Patient 33, preDM)

Patients living with T2DM and preDM talked about two specific diabetic complications they wanted to avoid: amputation and dialysis. These patients understood the potential long‐term complications of diabetes from observing people in their families and communities suffer. One patient used ‘loss’ as a euphemism to describe amputation: ‘I don't want to lose anything. It would really hurt me to lose something from this disease’. (Patient 8, T2DM) One patient who had worked in a long‐term care facility recounted, ‘Because of the area that I work in, I see how it can affect you if you do not take care of yourself. I do not want to be a dialysis patient. That would kill me because I like my freedom, and that is so restrictive. So I know the consequences if you don't behave’. (Patient 11, preDM)

For some patients, goals were connected to the ability to maintain relationships in the short‐ and the long‐term. One patient described ‘missing out’ on her grandchildren's lives, saying ‘I would go [visit them] like every other month if I was healthier’. (Patient 18, T2DM) Long‐term, patients did not want future health‐care demands to affect family relationships. One patient said, ‘I don't want to be burden to any of my family members’. (Patient 21, T2DM) These concerns extended even further to life expectancy relative to family members. One patient reported, ‘my husband's nine years younger than I am so I want to stick around as long as I can to enjoy our time together’. (Patient 24, T2DM)

In contrast to patients who perceived that diabetes can go away, these patients described improved numerical targets and goal achievements as diabetes that is ‘under control’. One patient described control as a goal: ‘I try to live healthier, move more, eat better and try to control it through diet’. (Patient 5, T2DM) Another patient described his understanding of the distinction between permanence and absence in terms of control: ‘the recent research says that once you enter the different zones that they consider prediabetes or diabetes they don't really take you out anymore. They consider you're not in remission, but under control’. (Patient 16, T2DM) One patient also talked about this ability to control preDM. One patient explained, ‘As long as I think I’m in control and it can be controlled, I have no fear. I’d say as long as I can stay under 5.5, I’m fine’. (Patient 12, preDM)

#### 3.1.2 TIMELINE 2: Can get rid of diabetes

Although patients used a variety of terms to describe it, a set of patients did have an expectation of reaching normoglycaemia. Some patients talked about it in terms of present versus absent. One patient described, ‘Well, I think you can get rid of diabetes’ (Patient 25, T2DM)

Other patients recognized the continuum nature of glycaemic control. One patient described moving back down the continuum: ‘You can move from one condition with the help of lifestyle changes and medication, you can move from the far extreme to the borderline to the all‐clear or the out‐of‐immediate‐danger area’. (Patient 22, T2DM) This idea of moving along a continuum was shared among patients who had been told they had preDM. Patients living with preDM recognized that it was a warning and that with behaviour change they could achieve normoglycaemia. One patient living with preDM recounted, ‘[doctor] said if I didn't watch myself, that I could end up on medication…[doctor] goes you really gotta change your lifestyle. You can stop it now while you're in the pre phase, or you can keep doing whatever you're doing and you can be diabetic, and then eventually you could be getting shots’. (Patient 13, preDM)

For both patients with T2DM and preDM, when patients talk about reaching normoglycaemia, it was linked to weight loss. ‘If my weight goes down to 190‐199, I won't have it (diabetes)’. (Patient 4, T2DM) This patient living with preDM reported a similar potential outcome: ‘[The doctor] told that if I lost the weight, then it would probably go back to no prediabetes, but just a regular person without diabetes’. (Patient 31, preDM)

Even though patients living with T2DM had not achieved it themselves, patients talked about normoglycaemia as a possibility. They connected this potential to unrealized weight loss. ‘If I lost… right now, if I lost forty pounds, I’d have no health problems. I’d have no health problems, according to the doctor. He said, you'd be cured’. (Patient 25, T2DM) In addition to weight loss, patients described surgical options as a method to achieve normoglycaemia. One patient reported, ‘At one time, I think it *may* have been that doctor even said something about here's some of the long‐term kind of stuff of, you know, take medication. You know, we've had people that have gotten gastric bypass and that like eliminates the diabetes’. (Patient 5, T2DM)

For patients living with preDM, they recognized that if they achieved normoglycaemia, they would need to maintain lifestyle modification. One patient whose bloodwork had improved described, ‘The last time they ran bloodwork on me, my doctor told me I’m doing real good. She says if I keep doing what I’m doing, we could say I’m not prediabetic based on the bloodwork, but I still have to be careful because I’m pre‐dispositioned for it. So I was like, so then what does that mean? She goes, well, that means you can't just go back to doing what you were doing before. You gotta keep doing what you're doing. This is a lifestyle change’. (Patient 13, preDM)

### 3.2 Phase 2: Physician practice through a quantitative approach

In the first case, which described a patient who met the clinical criteria for preDM at the first encounter, 136 (48.2%) of respondents would use the word ‘prediabetes’ to communicate the results to the patient, while 120 (42.3%) would say ‘at risk for diabetes’. In the follow‐up encounter, the patient had made successful lifestyle changes and experienced improved glycaemic control towards normoglycaemia. Of the 136 physicians who communicated preDM in the initial encounter, 53 (39.0%) would not use the words ‘prediabetes’ or ‘diabetes’ to explain the results, 31 (22.8%) would use risk for diabetes language, and the remaining 52 (38.2%) physicians would still communicate that the patient has preDM in some way.

In the second case, which described a patient who met the clinical criteria for T2DM, 282 physicians (98.6%) reported they would tell the patient that he has ‘diabetes’. In the follow‐up encounter, the patient had made successful lifestyle changes and experienced improved glycaemic control towards the prediabetes range. At this second appointment, of the 282 physicians who communicated diabetes in the first encounter, 267 (94.6%) would tell the patient he still had diabetes, 9 (3.2%) would communicate ‘the risk factor of diabetes’, and 6 (2.1%) would not use the words ‘prediabetes’ or ‘diabetes’ to communicate the results.

## 4 DISCUSSION

Findings here present dissonance in patient perceptions about the duration of diabetes and clinical practice. Though case reports of remission of T2DM have existed since at least 1953^46^ and recent studies demonstrate partial or complete remission of T2DM through intensive lifestyle interventions,^3^, ^4^, ^5^, ^6^, ^7^ physicians typically regard T2DM as a lifelong disease. Findings here provide a deeper understanding that echoes patient surveys that previously demonstrated patient beliefs that T2DM is not lifelong and able to be cured.^35^ However, quantitative findings here demonstrate that it is unclear that physicians share that perspective. Fairchild and colleagues suggest that ‘providers should educate patients on the natural history of diabetes’,^36^ but we also argue that physicians should have a clear, transparent conversation with patients to better appreciate each other's understanding of the diagnosis and each other's goals in clinical management of diabetes.^25^, ^47^

Through their communication about diabetes, physicians influence patient beliefs and ideas about illness,^48^ which have been associated with disease self‐management.^49^, ^50^, ^51^ In the clinical interaction, physicians are challenged to both present information and offer clear guidance on specific behaviours that the patient can enact to minimize health threats associated with diabetes.^48^ Research provides lessons for how to talk to patients about the diagnosis and treatment of T2DM based on an understanding of patient beliefs and ideas about their disease.^52^, ^53^, ^54^, ^55^ Motivational interviewing is one tool primary care physicians can use to counsel patients with preDM and T2DM,^28^ and this tactic is well aligned with the concept of movement along a continuum.

Our research team has previously demonstrated that patients perceive preDM as less ‘chronic’ than T2DM; patients living with preDM scoring it lower on the DIRQ timeline measure than patients living with T2DM.^40^ Our results provide a richer picture of that difference. Like patients diagnosed with T2DM, patients with preDM describe divergent views of the duration of the condition. Some patients perceived that preDM was a warning sign of what was ahead—that they could slow down or stop the process—whereas others saw it as a sign to turn around and head back to normoglycaemia. More research is needed to understand these different perceptions of the risk factor of preDM. It is possible that how physicians communicate that signpost can affect patient motivation to implement lasting lifestyle modification. Future inquiry should connect patient perceptions of the duration of diabetes to self‐management. This could indicate a need for patient education about the permanence of metabolic disorders. The PREPARE programme targeting patients with impaired glucose tolerance included timeline as a target for preDM education.^56^, ^57^

Survey results showed that more than 40% of our sample would not use the word ‘prediabetes’ in counselling patients regarding their diagnosis. These practice habits likely contribute to the fact that nearly 90% of adults in the United States who have preDM are unaware of their diagnosis.^58^ Alternately, this could reflect the lack of consensus regarding diagnostic thresholds for preDM^11^, ^59^ or the contested nature of the diagnosis of preDM itself.^60^, ^61^, ^62^ However, not informing patients of their diagnosis of preDM does not communicate the known risks associated with preDM, among them premature death,^63^, ^64^ coronary artery disease,^65^ transient ischaemic attack and stroke,^66^ and progression to T2DM and its associated complications.

Long‐term, large‐scale studies of intensive lifestyle intervention in people with preDM have demonstrated significant reductions in the rate of conversion to T2DM.^67^, ^68^, ^69^ Informing patients of their preDM also represents an opportunity to motivate patient behaviours early in the continuum of glucose tolerance, when remission is more likely.^5^, ^7^ Evidence also suggests that recommending these lifestyle changes leads to changed patient behaviours and increased physical activity in patients with preDM.^70^, ^71^, ^72^, ^73^

Although more vague terms such as ‘hyperglycemia’ and ‘impaired glucose tolerance’ may be used to communicate with patients, specifically naming the diagnosis influences patient representations of disease.^74^ We propose that using the term ‘prediabetes’ in the setting of motivational interviewing and risk communication is important to communicate a clear message and a path forward.

The vast majority of physicians in this sample would still say ‘diabetes’ to the patient who is working towards remission in the second case. Recent patient surveys that demonstrated patient beliefs that T2DM is not lifelong, is able to be cured, may not require medication when glucose levels are normal and may not require medications for life were presented as evidence that patients have ‘unrealistic expectations of treatment, as exemplified by the finding that one‐third expected their doctor to cure them of diabetes’,^35^ and that ‘providers should educate patients on the natural history of diabetes…’^36^ This result likely echoes the dominate physician perception of T2DM as a lifelong disease.

In line with a qualitative design, our goal was to engage in an exploratory study to better understand patient perceptions of the course of diabetes and their own goals of care. We interviewed patients living with preDM or T2DM, as patients living with these two conditions encounter similar information that may impact their self‐management behaviours. Still, future studies should explore differences in patients’ experiences based on living with preDM or T2DM. Although the number of subjects was relatively low, the group was diverse and the main themes reached saturation, suggesting that enough subjects were enrolled. In the quantitative phase, questions are subject to social desirability bias, such that the communication options chosen by the respondents may not accurately reflect clinical practice. A limitation of our study is that there is currently no consensus regarding the definition of remission of T2DM.^2^, ^8^ Physicians in our study, if aware of the possibility of remission, may follow the most stringent proposed measures of remission and therefore may not have considered the patient to have reach remission. Further study regarding physician knowledge, attitudes and beliefs about these concepts should be conducted to determine why physicians are not doing so.

## 5 CONCLUSION

Most physicians here elected to not communicate remission or regression in patient care. Yet, patients describe two different potential timelines for diabetes, including a subset of patients who expect their diabetes can be ‘cured’. Physicians should incorporate shared decision making to create a shared mental model of diabetes and its potential outcomes with patients.

## 6 DISCLAIMER

The views expressed within this publication represent those of the authors and do not reflect the official position of the US Air Force, US Army, Henry M. Jackson Foundation, Uniformed Services University of the Health Sciences or the US Government, the Department of Defense at large.

## AUTHORS’ CONTRIBUTION

Drs. Ledford, Fulleborn, Rogers and Samar contributed to the study design and implementation as well as drafting the manuscript. Mr Jackson assisted in the writing and editing of the manuscript. All authors read and approved the final manuscript.

## Data Availability

Transcribed data are not available for release.
